# Spondyloarthritis-related and degenerative MRI changes in the axial skeleton - an inter- and intra-observer agreement study

**DOI:** 10.1186/1471-2474-14-274

**Published:** 2013-09-23

**Authors:** Bodil Arnbak, Tue Secher Jensen, Claus Manniche, Anna Zejden, Niels Egund, Anne Grethe Jurik

**Affiliations:** 1 Research Department, Spine Centre of Southern Denmark, Hospital Lillebaelt, Oestre Hougvej 55, Middelfart 5500, Denmark; 2 Institute of Regional Health Services Research, University of Southern Denmark, Winsloewparken 19, Odense C 5000, Denmark; 3 Department of Radiology, Aarhus University Hospital, Aarhus Sygehus, Noerrebrogade 44, 8000, Aarhus C, Denmark

**Keywords:** Agreement, Ankylosing spondylitis, Arthritis, Diagnosis, Kappa, Low back pain, Magnetic resonance imaging, Sacroiliac joint, Sacroiliitis, Spine, Spondyloarthropathy, Spondylarthritis

## Abstract

**Background:**

The Back Pain Cohort of Southern Denmark (BaPa Cohort) was initiated with the aim of evaluating the clinical relevance of magnetic resonance imaging (MRI) in the diagnosis of early spondyloarthritis (SpA). In order to facilitate the collection of MRI data for this study, an electronic evaluation form was developed including both SpA-related and degenerative axial changes. The objective of the current study was to assess the intra- and inter-observer agreement of the MRI changes assessed.

**Methods:**

Three radiologists evaluated 48 MRI scans of the whole spine and the sacroiliac joints from a subsample of the BaPa Cohort, consisting of patients with non-specific low back pain and patients with different stages of SpA features. The spine was evaluated for SpA-related and degenerative MRI changes and the SIJ for SpA-related changes. Inter- and intra-observer agreements were calculated with kappa statistics. In the interpretation of the kappa coefficient, the standards for strength of agreement reported by Landis and Koch were followed.

**Results:**

A total of 48 patients, 40% men and mean age of 31 years (range 18 – 40 years), were evaluated once by all three readers and re-evaluated by two of the readers after 4-12 weeks. For MRI changes in the spine, substantial to almost perfect observer agreement was found for the location and the size of vertebral signal changes and for disc degeneration and disc contour. For the sacroiliac joints, substantial or almost perfect observer agreement was found for the grading of bone marrow oedema and fatty marrow deposition, the depth of bone marrow oedema and for subchondral sclerosis. Global assessment of the SpA diagnosis had substantial to almost perfect observer agreements.

**Conclusion:**

The acceptable agreement for key MRI changes in the spine and sacroiliac joints makes it possible to use these MRI changes in the BaPa Cohort study and other studies investigating MRI changes in patients with non-specific low back pain and suspected SpA.

## Background

Spondyloarthritis (SpA) is a group of rheumatological disorders, which result in back pain, and stiffness of the spine due to inflammatory and structural changes in the spine and the sacroiliac joints (SIJ). Plain-film radiography can detect structural changes but not early inflammatory changes. Magnetic resonance imaging (MRI) has been reported to identify both structural and inflammatory changes [1,2] and is considered essential in the diagnoses of SpA. However, there are still several uncertainties regarding the utility of MRI in the diagnosis of SpA [3], especially in the early stages when the clinical signs of SpA can be difficult to distinguish from non-specific low back pain (LBP) and the MRI signs of SpA can be difficult to distinguish from the much more common findings of degeneration. Signal changes related to degeneration such as Modic changes are an important pitfall in the assessment of SpA [4] and some studies have shown substantial variation in the extent of MRI lesions in the SIJ previously considered to be specific for SpA [5]. Therefore, studies encompassing patients reflecting the target population and using a MRI protocol including both SpA-related and degenerative changes are needed to validate the utility of this new imaging modality for the diagnosis of SpA.

On this basis, the Back Pain Cohort of Southern Denmark (BaPA Cohort) was initiated in 2011 at the Spine Centre of Southern Denmark with the aim of evaluating the clinical relevance of MRI in the diagnosis of early SpA. In order to facilitate the quantification of MRI changes in detail, an electronic evaluation form was developed for the evaluation of SpA-related and degenerative changes in the spine and SpA-related changes in the SIJ. The electronic MRI evaluation protocol was based on existing grading systems of active and chronic SpA changes in the spine [6] and SIJ [7]. These grading systems have been tested for inter- and intra-observer agreement in sum-scores with good results [6,7]. However, the current evaluation form was more detailed and included both SpA-related and degenerative spinal MRI changes. Thus, a new assessment of observer agreement was required.

The objective of the current study was therefore to assess the intra- and inter-observer agreement of SpA-related and degenerative changes in the spine and SpA-related changes in the SIJ assessing each lesion separately.

## Methods

### The study population

The analysis encompassed 48 sets of whole spine MRI scans in addition to MRI of the SIJ. All MRI scans were acquired from a subset of patients (n = 350) of the BaPa Cohort enrolled between March 2011 and February 2012. The BaPa Cohort consists of randomly selected patients aged between 18 and 40 years, referred to a secondary care sector outpatient spine clinic (Spine Centre of Southern Denmark). Patients were referred to the Centre for episodes of LBP ranging from 2 to 12 months, where there had been insufficient effect following conservative treatment in the primary care sector and there was no suspicion of specific LBP conditions such as SpA, fracture, cancer or infection. All patients who were included in the BaPa cohort received an MRI scan of the whole spine and the SIJ.

The patients included in the current analysis were selected by the primary investigator (BA) without involvement of the evaluating radiologists. Due to the low prevalence of some MRI changes to be evaluated in this cohort, 38 patients were chosen based on data from previous systematic evaluations of the MRI scans. The previous systematic evaluations were done at least 4 months prior to the readings in the current study. This selection method was used to increase the number of ‘positive’ MRI changes, thereby ensuring sufficient statistical power to calculate reliable kappa values. The remaining 10 patients were randomly selected from the remaining 312 patients.

### Magnetic resonance imaging technique and evaluation

MRI of the whole spine and the SIJ was performed with a 1.5 T Philips Achieva (Best, The Netherlands) MRI System. A SENSE spine coil was used for imaging with the study participants in the supine position. The whole spine sequences were performed in three steps (cervical, thoracic and lumbar) subsequently fused digitally and encompassing:

•Sagittal short-tau inversion recovery (STIR): time to repeat (TR)/ time to echo (TE)/ time to inversion (TI) 2500/60/170 ms and 2 acquisitions; matrix 320 × 231, field of view (FOV) 300 × 300 mm, 16 slices with a thickness of 4 mm and interslice distance 1 mm; scan time 1 min 55 s.

•Sagittal T1-weighted turbo spin echo: TR/TE 475/12 ms and 2 acquisitions; matrix 336 × 252, FOV 336 × 252 mm, 16 slices with a thickness of 4 mm and interslice distance 1 mm; scan time 2 min 3 s.

•Additionally for the lumbar spine: Sagittal T2-weighted VISTA (3D – turbo spin echo-T2-weighted sequence): TR/TE 2000/120 ms and 2 acquisitions; matrix 324 × 148, FOV 182 × 325 mm, 73 slices with a thickness of 1 mm; scan time 6 min 22 s. 3D reconstruction was not used in the current study.

For the SIJ the following sequences were used:

•Semicoronal T1-weighted turbo spin echo: TR/TE 535/14 ms and 4 acquisitions; matrix 512 × 255, FOV 300 × 300 mm, 18 slices with a thickness of 4 mm and interslice distance 0.4 mm; scan time 5 min 36 s.

•Semicoronal T1-weighted Spectral Pre-saturation with Inversion Recovery (SPIR): TR/TE 525/8 ms and 4 acquisitions; matrix 200 × 274, FOV 343 × 180 mm, 18 slices with a thickness of 4 mm and interslice distance 0.4 mm; scan time 4 min 53 s.

•Semiaxial STIR long TE: TR/TE/TI 3500/60/155 ms and 8 acquisitions; matrix 500 × 153, FOV 250 × 205 mm, 22 slices of 4 mm thickness and interslice distance 0.4 mm; scan time 8 min 24 s scan time.

The images were read on dedicated radiological workstations with two 21-inch high-resolution screens. All MRIs were anonymised and blinded for all clinical information including previous readings and the patient’s age and gender.

Three observers evaluated the images independently. They were all senior consultant radiologists at the Department of Radiology, Aarhus University Hospital, and were specialised in musculoskeletal imaging and SpA. Prior to the study, two calibration sessions were conducted. After a period of 4-12 weeks, two observers (AJ and AZ) re-evaluated all 48 MRI scans for intra-observer agreement.

**Figure 1:**
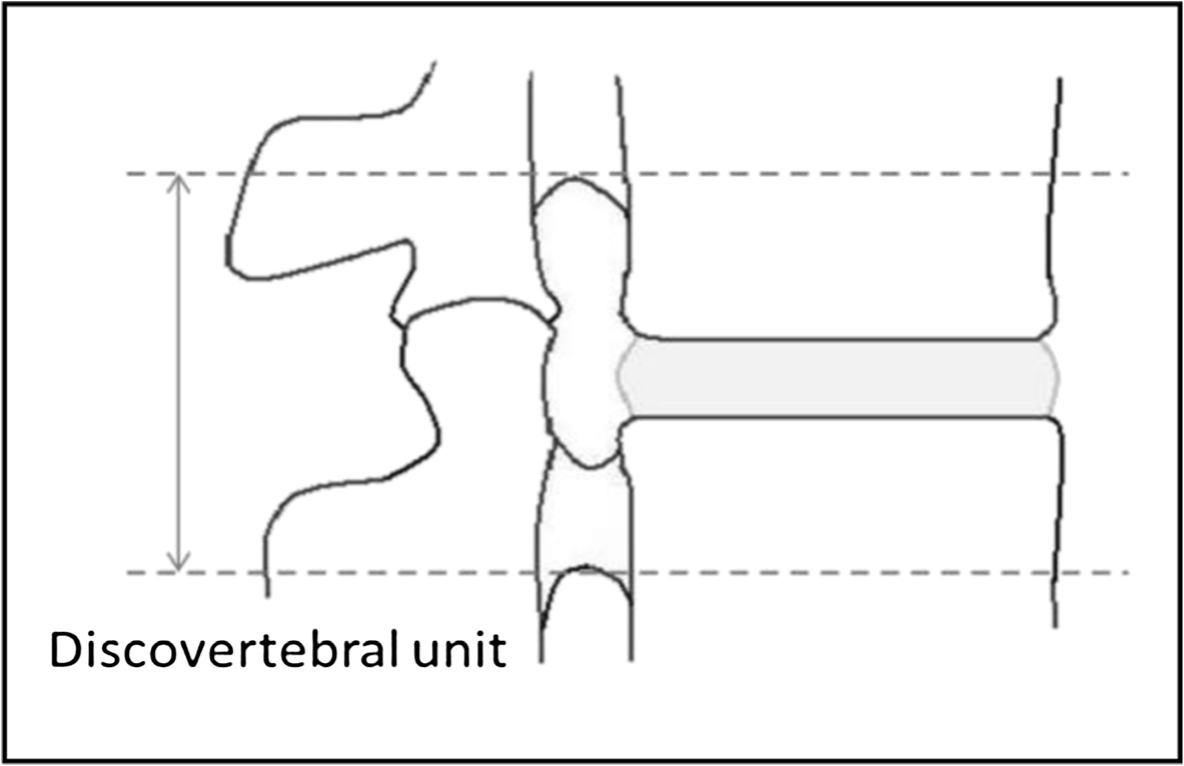
**Discovertebral unit modified from **[6]**.**

The evaluation form consists of two parts: 1) evaluation of the spine and 2) evaluation of the SIJ. The spine was divided in 23 disco-vertebral units (DVU) from C2-C3 to L5-S1. A DVU was defined as the region between two virtual horizontal lines through the centre of two adjacent vertebrae (Figure 1). Furthermore, each vertebral endplate and subjacent bone marrow area of a DVU were assessed separately for variables related to signal changes or erosions. An estimate of the total vertebral endplate and subchondral bone marrow areas was based on all sagittal slices creating “3D like picture” of the changes. The spinal MRI changes assessed are listed in Table 1. For a detailed definition of the MRI changes assessed, see Additional file 1.

**Table 1:** Grading of MRI changes in the spine

**Name of the MRI changes**	**Grading**
Type of signal change	BMO
	FMD
	Mixed
*Signal change in the corner*^*1*^	Yes/No
*Location of signal change*	Anterior
	Posterior
	Equally widespread
*Size of signal change*	Small
	Medium
	Large
Total size of BMO in the DVU	Small
	Medium
	Large
Total size of FMD in the DVU	Small
	Medium
	Large
Total size of mixed lesions in the DVU	Small
	Medium
	Large
Erosions	None
	Slight
	Moderate
	Severe
Erosion of the corner^1^	Yes/No
Syndesmophytes or vertebral fusion	None
	Syndesmophytes between corners
	Partial osseous bridging
	Total fusion
BMO at the apophyseal joints	Yes/No
BMO at the costovertebral joints	Yes/No
FMD at the apophyseal joints	Yes/No
Soft tissue oedema	Yes/No
Disc degeneration	Normal height and signal intensity
	Slightly decrease in height and signal intensity
	Decreased height and fluid signal
	Elimination of the disc height
Disc contour	Broad-based protrusion
	Focal protrusion
	Extrusion
	Sequestration (free fragment)
Disc herniation in the vertebral endplate	Yes/No
Scheuermann’s changes	Yes/No

BMO: Bone marrow oedema, FMD: fatty marrow deposition, DVU: discovertebal unit, Small/Slight: < 25% of the subcortical bone area, Medium/Moderate: 25% - <50% of the subcortical bone area, Large/Severe: ≥50% of the subcortical bone area.

The SIJs were subdivided in four osseous locations for each joint: the iliac and sacral bone corresponding to the cartilaginous and the ligamentous portion of the joint, respectively. An estimate of the total cartilaginous and ligamentous joint facets and the adjacent subchondral bone marrow areas was based on all semicoronal and semiaxial slices creating a 3D picture of both joint portions. The MRI changes assessed at the SIJ are listed in Table 2 according to the Danish method described previously [7]. For a detailed definition of the MRI changes assessed, see Additional file 1.

**Table 2:** Grading of MRI changes at the sacroiliac joints and global assessment

**Name of the MRI changes**	**Grading**
BMO	Slight
	Moderate
	Severe
*Intensity of BMO*	Normal or slightly increased intensity
	Pronounced increased
*Depth of BMO*	Normal to moderate depth
	Pronounced
FMD	Slight
	Moderate
	Severe
*Depth of FMD*	Normal to moderate
	Pronounced
Erosions	Slight
	Moderate
	Severe
Subchondral sclerosis	Slight
	Moderate
	Severe
Ankylosis	Partial
	Total
Global assessment: ‘This patient has SpA’^1^	Strongly disagree
	Disagree
	Neither agree or disagree
	Agree
	Strongly agree

BMO: Bone marrow oedema, FMD: fatty marrow deposition, Pronounced increased: Signal intensity comparable with that of the spinal fluid and covering an area of ≥1 cm^2^. Pronounced: Oedema extending ≥1 cm beneath the joint surface and covering ≥1 cm^2^. Slight: < 25% of the subcortical bone area, Moderate: 25% - <50% of the subcortical bone area, Severe: ≥50% of the subcortical bone area. ^1^Assessed on the entire MRI examination, not only the SIJ.

Global assessment of the SpA diagnosis was based on MRI changes in both the spine and the SIJ. Both regions were assessed at the same session. For each patient, the observer was asked to rate how strongly he/she agreed with the following: ‘This patient has SpA’. For a detailed definition of the MRI changes assessed, see Additional file 1.

In the statistical analysis, the number of observations varied according to the variables assessed. In the spine, variables related to signal changes (with the exception of the total size of the signal changes) or erosions were evaluated for both the upper and lower endplates of 23 DVUs in the 48 patients (2208 endplates). ‘Bone marrow oedema (BMO) in the costovertebral joints’ was evaluated at 12 vertebral levels in 48 patients (576 levels). The remaining spinal variables were evaluated in 23 DVUs from 48 patients (1104 DVUs). In the SIJ, 8 regions in the 48 patients were evaluated (384 regions).

### Data entry

The data were entered directly into a comprehensive clinical and imaging electronic database (the SpineData database) using an internet-based evaluation form. Data were subsequently exported to, and stored in, STATA11 format (StataCorp, 2000, *Stata Statistical Software: Release 11.2*, College Station, TX: STATA Corporation, USA) and checked for logic and consistency using the STATA ‘do files’ as documentation.

### Statistical analysis

To assess the inter- and intra-observer agreement, ratings from each observer were cross-tabulated and agreement was measured using kappa statistics [8]. Results were reported as observed agreement, expected agreement and kappa values with 95% confidence intervals (CI) for each pair of observers and combined for all three observers.

Kappa is defined as the difference between observed and expected agreement (by chance), expressed as a fraction of the maximum difference. Kappa = (observed agreement - expected agreement) / (1 - expected agreement) [8]. Dichotomous and nominal categorical variables were tested with ordinary kappa statistics and ordered categorical variables were tested with weighted Kappa. Quadratic weights were applied according to the number of categories. The quadratic weights are specified as 1 - {(i-j)/(k-1)}^2 where i and j index the rows and columns of the ratings by the two readers and k is the number of categories. The intra-class correlation coefficient (ICC), which is similar to an overall quadratic weighted kappa [9], was used as a measure of overall agreement between the three observers with the exception of two nominal categorical variables which had more than two categories: ‘type of signal change’ and ‘location of signal change in the vertebral endplate’ which were analysed with ordinary kappa. ICC was tested in a one way ANOVA model (absolute agreement).

95% confidence interval (CI) was calculated with an analytical method in the case of dichotomous variables [10] and by bootstrap resampling with 3000 repetitions for categorical variables with more than two categories [11,12].

In the interpretation of the kappa coefficient the standards for strength of agreement given by Landis and Koch were followed defined as slight (κ < 0.2), fair (0.2 ≤ κ < 0.4), moderate (0.4 ≤ κ < 0.6), substantial (0.6 ≤ κ < 0.8) and almost perfect (0.8 ≤ κ < 1) [13].

Only endplates where both readers agreed on the presence of a signal change were included in the analyses for the following variables: ‘Signal change in the corner’, ‘location of signal change in the vertebral endplate’, and ‘size of signal change’, so the statistical analysis was a measure of agreement of location and size and not the presence of the given signal change. Similarly, only endplates where both readers agreed on the presence of erosions were included in the analyses for ‘erosions in the corner’. In relation to intensity and depth of BMO and the depth of fatty marrow deposition (FMD), only observations where both readers agreed on the presence of BMO or FMD respectively, were included in the analyses.

Analogous to the requirements for valid inference for contingency tables, we used a criterion of having at least 5 positive ratings for each variable for inclusion in the kappa analyses.

For statistical analysis, the STATA11 statistical package was used.

### Ethics

The project was conducted in accordance with the Helsinki-II declaration. The Regional Scientific Ethical Committee for Southern Denmark has evaluated the study as not obligated of notification. Each patient gave written informed consent for research use and publication of their data. The establishment of the database is registered at the Danish Data Protection Agency and all clinical information about the participants are kept confidential and in line with the Danish Act on Processing of Personal Data.

## Results

A total of 48 patients, 40% men and a mean age of 31 years (range 18 – 40 years), were evaluated once by all three readers and re-evaluated by two of the readers after 4-12 weeks.

### Spinal MRI changes

In relation to the combined inter-observer agreement of the spinal MRI changes, four findings: ‘erosion of the corner’, ‘BMO at the costovertebral joints’, ‘FMD at the apophyseal joints’ and, ‘soft tissue oedema’, were excluded because of too few positive ratings (Table 3).

**Table 3:** Prevalence of positive ratings for the MRI changes assessed

	**Name of the MRI changes**	**Reader A, first reading**	**Reader B, first Reading**	**Reader C, first reading**	**Reader A2, second reading**	**Reader B2, second reading**
**Spine**	Type of signal change	114	147	88	118	154
	Signal change in the corner^1^	38	57	13	39	47
	Location of signal change^1^	114	147	88	118	154
	Size of signal change^1^	114	147	88	118	154
	Total size of BMO lesions in DVU	36	32	11	12	31
	Total size of FMD lesions in DVU	38	39	32	14	41
	Total size of mixed lesions in DVU	9	25	10	4	27
	Erosions	3	16	11	3	17
	Erosion of the corner	1	3	0	0	0
	Syndesmophytes or vertebral fusion	5	8	3	7	6
	BMO at apophyseal joints	8	4	5	10	5
	BMO at costovertebral joints	1	4	1	1	4
	FMD at apophyseal joints	4	0	1	1	3
	Soft tissue oedema	1	0	0	0	0
	Disc degeneration	160	149	109	150	153
	Disc contour	122	101	99	111	105
	Herniation in the vertebral endplate	23	19	6	21	27
	Scheuermann’s changes	12	5	15	9	10
**SIJ**	BMO	71	77	58	70	85
	Intensity of BMO^1^	15	6	0	13	7
	Depth of BMO^1^	27	21	24	27	30
	FMD	59	84	60	62	80
	Depth of FMD^1^	36	44	48	33	42
	Erosions	32	39	9	35	43
	Subchondral sclerosis	19	21	9	15	21
	Ankylosis	4	11	8	4	8

The numbers in the table refer to ratings > 0 (not normal) for each of the MRI changes. Only MRI changes with at least 5 positive ratings pr. reading were included in the kappa analyses. BMO, Bone marrow oedema; FMD, Fatty marrow deposition. ^1^Only observations where the reader on both the readings agreed on the presence of a signal change, BMO or FMD, respectively, were kept in the analysis.

The strength of the combined inter-observer agreement for spinal MRI changes ranged from slight (κ = .12) to almost perfect (κ = .90). Almost perfect agreement was found for ‘location of signal changes in the vertebral endplate’. Substantial agreement was found for ‘size of signal change’, ‘disc degeneration’ and ‘disc contour’. Moderate agreement was found for ‘type of signal change’, ‘signal change in the corner’, ‘total size of FMD lesions in the DVU’ and ‘herniation in the vertebral endplate’. Fair agreement was found for the ‘total size of BMO in the DVU’ and ‘total size of mixed lesions in the DVU’. Slight agreement was found for ‘Scheuermann’s changes’. For ‘erosions’, ‘syndesmophytes or vertebral fusion’ and ‘BMO at the apophyseal joint’, only single pairwise analyses of inter-observer agreement were possible because of too few positive ratings. These analyses showed a fair, moderate and moderate agreement, respectively (Table 4).

**Table 4:** Inter-observer agreements for spinal MRI changes

	**Observers**	**Observed agreement (%)**	**Expected agreement (%)**	**Pairwise weighted Kappa (95% CI)**	**Number of levels**^**1**^
Type of signal change	AB	95.70	88.65	.62 (.56–.68)	2208
	AC	96.38	91.13	.59 (.51–.66)	2208
	BC	95.70	89.73	.58 (.51–.65)	2208
	Combined			.60 (.55–.65)	2208
*Signal change in the corner*^*2*^	AB	83.51	56.32	.62 (.46–.79)	97
	AC	80.00	61.22	.48 (.28–.69)	70
	BC	72.15	64.01	.23 (.00–.46)	79
	Combined^3^			.53 (.42–.64)	114
*Location of signal change*^*2*^	AB	92.78	42.56	.87 (.78–.95)	97
	AC	97.14	45.31	.95 (.86–1.00)	70
	BC	94.94	46.37	.91 (.79–.98)	79
	Combined			.90 (.83–.96)	66
*Size of signal change*^*2*^	AB	91.49	75.74	.65 (.45–.80)	97
	AC	93.57	74.92	.74 (.54–.89)	70
	BC	97.61	90.05	.76 (.64–.86)	79
	Combined^3^			.66 (.58–.75)	114
Total size of BMO in the DVU	AB	99.41	98.71	.54 (.32–.74)	1104
	AC	99.23	99.07	.16 (.06–.33)	1104
	BC	99.62	99.40	.43 (.24–.63)	1104
	Combined^3^			.38 (.34–.41)	1104
Total size of FMD in the DVU	AB	99.09	98.12	.52 (.36–.66)	1104
	AC	99.25	98.27	.57 (.41–.72)	1104
	BC	99.12	98.31	.47 (.32–.63)	1104
	Combined^3^			.52 (.49–.56)	1104
Total size of mixed lesions in the DVU	AB	99.39	98.99	.39 (.12–.65)	1104
	AC	99.50	99.42	.13 (.00–.36)	1104
	BC	99.59	99.19	.49 (.22–.74)	1104
	Combined^3^			.36 (.32–.40)	1104
Erosions	AB	-	-	-	-
	AC	-	-	-	-
	BC	99.83	99.72	.40 (.17–.56)	2208
	Combined^3^				-
Syndesmophytes or vertebral fusion	AB	99.71	99.17	.65 (0.00–.91)	1104
	AC	-	-	-	-
	BC	-	-	-	-
	Combined^3^			-	-
BMO at the apophyseal joint	AB	-	-	-	-
	AC	99.55	98.83	.61 (.30–.93)	1104
	BC	-	-	-	-
	Combined^3^			-	-
Disc degeneration	AB	98.61	94.73	.74 (.67–.79)	1104
	AC	98.91	94.98	.78 (.72– .84)	1104
	BC	98.97	95.61	.77 (.70–.82)	1104
	Combined^3^			.76 (.74–.78)	1104
Disc contour	AB	98.55	95.17	.70 (.63–.76)	1104
	AC	98.44	94.29	.73 (.65–.79)	1104
	BC	97.36	91.96	.67 (.60–.74)	1104
	Combined^3^			.70 (.68–.73)	1104
Herniation in the vertebral endplate	AB	98.73	96.27	.66 (.49–.83)	1104
	AC	98.10	97.40	.27 (.06–.48)	1104
	BC	98.28	97.75	.23 (.01–.46)	1104
	Combined^3^			.43 (.40–.47)	1104
Scheurmann’s changes	AB	98.46	98.47	-.01 (-.01–0.00)	1104
	AC	98.92	97.58	.14 (-.05–.32)	1104
	BC	98.55	98.20	.20 (-.04–.43)	1104
	Combined^3^			.12 (.08–.15)	1104

BMO; Bone marrow oedema, DVU; Discovertebral unit, FMD; Fatty marrow deposition, - Too few positive ratings for one of the observers to be included in the analysis ^1^Discovertebral units or vertebral endplates. ^2^Only observations where both readers agreed on the presence of a signal change were included in the analyses. ^3^Intraclass correlation coefficient.

In relation to the intra-observer agreement, four MRI findings: ‘erosions in the corner’, ‘BMO at the costovertebral joints’ ‘FMD at the apophyseal joints’ and ‘soft tissue oedema’ were excluded because of too few positive ratings. Furthermore, ‘erosions’ and ‘BMO at the apophyseal joint’ could only be analysed for one reader because of too few positive ratings (Table 3).

The strength of the intra-observer agreement for the spinal MRI changes ranged from moderate (κ = .56) to almost perfect (κ = .98) for reader A and from substantial (κ = .67) to almost perfect (κ = .93) for reader B. In general, the strength of intra-observer agreement was notably higher than the strength of inter-observer agreement (Table 5). All kappa values were above 0.7, except for two MRI changes for reader A (‘total size of BMO in the DVU’ and ‘total size of FMD in the DVU’) and one finding for reader B (‘Scheuermann’s changes’).

**Table 5:** Intra-observer agreements for spinal MRI changes

**MRI changes**	**Observers**	**Observed agreement (%)**	**Expected agreement (%)**	**Pairwise weighted Kappa (95% CI)**	**Number of levels**^**1**^
Type of signal change	AA	98.32	89.88	.84 (.78–.88)	2208
	BB	97.96	87.00	.84 (.80–.88)	2208
*Signal change in the corner*^*2*^	AA	89.22	54.61	.76 (.63–.90)	102
	BB	87.31	54.81	.72 (.60–.84)	134
*Location of signal change*^*2*^	AA	99.02	42.43	.98 (.94–1.00)	102
	BB	96.27	43.54	.93 (.87–.99)	134
*Size of signal change*^*2*^	AA	99.02	87.80	.92 (.85–.97)	102
	BB	98.18	91.18	.79 (.64–.91)	134
Total size of BMO in the DVU	AA	99.51	98.89	.56 (.23–.78)	1104
	BB	99.76	99.16	.71(.48–.88)	1104
Total size of FMD in the DVU	AA	99.43	98.64	.58 (.39–.74)	1104
	BB	99.81	99.12	.78 (.67–.87)	1104
Total size of mixed lesions in the DVU	AA	99.83	99.43	.71 (0.00–.95)	1104
	BB	99.61	98.52	.73 (.50–.88)	1104
Erosions	AA	-	-	-	-
	BB	99.91	99.61	.77 (.47–.91)	2208
Syndesmophytes or vertebral fusion	AA	99.95	99.19	.94 (.67–1.00)	1104
	BB	99.93	99.57	.83 (.40–.98)	1104
BMO at the apophyseal joint	AA	99.82	98.38	.88 (.73–1.00)	1104
	BB	-	-	-	-
Disc degeneration	AA	99.58	94.43	.92 (.89–.95)	1104
	BB	99.55	95.35	.90 (.87–.93)	1104
Disc contour	AA	99.26	94.28	.87 (.82–.91)	1104
	BB	99.25	93.36	.89 (.84–.93)	1104
Herniation in the vertebral endplate	AA	99.82	96.09	.95 (.89–1.00)	1104
	BB	98.91	95.92	.73 (.59–.88)	1104
Scheurmann’s changes	AA	99.55	98.12	.76 (.56–.96)	1104
	BB	99.55	98.65	.67 (.39–.94)	1104

BMO; Bone marrow oedema, DVU; Discovertebral unit, FMD; Fatty marrow deposition, - Too few positive ratings for one of the observers to be included in the analysis ^1^Discovertebral units or vertebral endplates. ^2^Only observations where both readers agreed on the presence of a signal change were kept in the analysis.

### Changes in the sacroiliac joints

The strength of the combined inter-observer agreement for evaluation of the SIJ changes ranged from moderate (κ = .52) to almost perfect agreement (κ = .81) (Table 6). Almost perfect agreement was found for ‘BMO’ and substantial agreement was found for ‘depth of BMO’, ‘FMD’ and ‘subchondral sclerosis’. Moderate agreement was found for ‘depth of FMD’ and ‘erosions’. For ‘intensity of BMO’ and ankylosis, only single pairwise analyses was possible because of too few positive ratings (Table 3). These analyses showed moderate and substantial agreement, respectively.

**Table 6:** Inter-observer agreements for MRI changes in the SIJ and global assessment

**MRI changes**	**Observers**	**Observed agreement (%)**	**Expected agreement (%)**	**Pairwise weighted Kappa (95% CI)**	**Number of SIJ regions**
BMO	AB	97.77	89.52	.79 (.69–.86)	384
	AC	98.09	91.08	.79 (.68–.87)	384
	BC	98.70	91.38	.85 (.79–.90)	384
	Combined^1^			.81 (.78–.84)	384
*Intensity BMO*^*2*^	AB	85.00	72.22	.46 (.19–.73)	60
	AC	-	-	-	-
	BC	-	-	-	-
	Combined^1^			-	-
*Depth BMO*^*2*^	AB	85.00	53.67	.68 (.49–.87)	60
	AC	82.35	50.52	.64 (.43–.85)	51
	BC	92.86	53.06	.85 (.71–.99)	56
	Combined^1^			.73 (.63–.82)	65
FMD	AB	97.25	83.26	.84 (.77–.89)	384
	AC	97.19	87.28	.78 (.69–.85)	384
	BC	95.60	84.77	.71 (.60–.80)	384
	Combined^1^			.78 (.74–.81)	384
*Depth FMD*^*2*^	AB	84.91	56.14	.66 (.44–.87)	53
	AC	78.05	63.65	.40 (.08–.71)	41
	BC	72.55	58.94	.33 (.07–.60)	51
	Combined^1^			.52 (.38–.67)	67
Erosions	AB	97.77	92.99	.68 (.49–.81)	384
	AC	98.32	96.12	.57 (.30–.76)	384
	BC	96.90	94.69	.42 (.20–.63)	384
	Combined^1^			.57 (.51–.62)	384
Subchondral sclerosis	AB	98.73	96.29	.66 (.40–.81)	384
	AC	99.16	97.21	.70 (.37–.87)	384
	BC	98.87	97.15	.60 (.28–.81)	384
	Combined^1^			.65 (.61–.70)	384
Ankylosis	AB	-	-	-	-
	AC	-	-	-	-
	BC	99.28	97.26	.74 (35–.90)	384
	Combined^1^			-	-
Global assessment^3^	AB	94.2	81.31	.69 (.44–.86)	47^4^
	AC	90.75	74.95	.59 (.35–.75)	44^4^
	BC	89.39	74.64	.58 (.32–.78)	43^4^
	Combined^1^			.61 (.46–.75)	47^4^

BMO: Bone marrow oedema, FMD: fatty marrow deposition, - Too few positive ratings for one of the observers to be included in the analysis ^1^ Intraclass correlation coefficient. ^2^Only observations where both readers agreed on the presence of BMO/FMD were kept in the analysis. ^3^Assessed on the entire MRI scan. ^4^Number of patients assessed.

The strength of intra-observer agreement was stronger than the inter-observer agreement. For reader A, the strength of agreement ranged from substantial (κ = .77) to almost perfect (κ = .96) and for reader B also from substantial (κ = .75) to almost perfect (κ = .91). For details, see Table 7.

**Table 7:** Intra-observer agreements for MRI changes in the SIJ and global assessment

**MRI changes**	**Observers**	**Observed agreement (%)**	**Expected agreement (%)**	**Weighted Kappa (95% CI)**	**Number of SIJ regions**
BMO	AA	99.57	89.42	.96 (.92–.98)	384
	BB	98.99	89.19	.91 (.85–.95)	384
*Intensity of BMO*^*1*^	AA	92.42	67.36	.77 (.58–.96)	66
	BB	95.95	83.97	.75 (.47–1.00)	74
*Depth of BMO*^*1*^	AA	95.45	51.93	.91 (.80–1.00)	66
	BB	91.89	55.84	.82 (.68–.96)	74
FMD	AA	99.33	85.49	.95 (.92–.97)	384
	BB	98.06	82.15	.89 (.84–.93)	384
*Depth of FMD*^*1*^	AA	95.57	52.40	.80 (.64–.97)	53
	BB	87.88	52.25	.75 (.58–.91)	66
Erosions	AA	98.61	93.52	.79 (.60–.92)	348
	BB	99.02	91.96	.88 (.80–.93)	384
Subchondral sclerosis	AA	99.62	96.84	.88 (.71–.96)	384
	BB	99.62	96.48	.89 (.77–.95)	384
Ankylosis	AA	-	-	-	-
	BB	99.54	97.26	.83 (.52–.95)	384
Global assessment^2^	AA	98.37	84.61	.89 (.82–.95)	46^3^
	BB	95.79	80.20	.79 (.52–.93)	46^3^

SIJ: sacroiliac joints, BMO: Bone marrow oedema, FMD: fatty marrow deposition, - Too few positive ratings for one of the observers to be included in the analysis ^1^Only observations where the reader on both the readings agreed on the presence of BMO/FMD were kept in the analysis. ^2^Assessed on the entire MRI scan, not only the SIJ. ^3^Number of patients assessed.

### Global assessment

The combined inter-observer agreement for ‘global assessment’ was substantial (κ = .61) (Table 6), whereas the intra-observer agreement was almost perfect (κ = .89) for reader A and substantial (κ = .79) for reader B (Table 7).

## Discussion

In this study, the agreement of different SpA-related and degenerative changes in the spine and SpA-related changes in the SIJ were tested jointly in a sample of patients with non-specific LBP only and patients with LBP associated with different stages of SpA. The majority of earlier studies on agreement on SpA-related and degenerative changes have been focused on separate regions of the spine, primarily the lumbar spine, whereas this study included the whole spine and the SIJ. Moreover, for the MRI changes evaluated in the SIJ, this is the first time agreement has been tested assessing each lesion separately.

In general, the agreement ranged from slight to almost perfect. As expected, the level of intra-observer agreement was higher than the inter-observer agreement. Agreements for MRI changes in the SIJ were generally stronger than for the spine. For the spinal MRI changes, ‘disc degeneration’ and ‘disc contour’ yielded the highest level of agreement followed by ‘location of signal changes in the vertebral endplate’, ‘size of signal change’ and ‘type of signal change’. In relation to the evaluation of the SIJ, ‘BMO’, ‘depth of BMO’, ‘FMD’, and ‘ankylosis’ were the changes with the best agreement. Global assessment showed substantial to almost perfect agreements.

The tendency of better reliability of the SpA-related findings in the SIJ compared to the spine could be explained by low prevalence of SpA-related findings in the spine. In addition, changes in the posterior spinal elements often are relatively small and can be difficult to assess on sagittal MRI slices.

### Comparison with previously published studies

The number of previous studies on observer agreements on spinal MRI changes related to SpA is limited. One previous study evaluated the agreement of structural SpA-related changes at each vertebral level in 20 patients with established SpA [14]. Kappa value of 0.60, 0.21, and 0.59 were found for ‘non-corner vertebral endplate erosions’, ‘vertebral corner spurs’ and ‘ankylosis’, respectively. However, differences in the definitions and in the study sample preclude a direct comparison with our results. Furthermore, there are published studies evaluating the agreement of sum scores for the whole spine [6,15], which unfortunately preclude comparison with the evaluation of changes at the endplate level.

In relation to the evaluation of signal changes in the endplates, these changes are not only observed in patients with suspected SpA but also in other populations. Several authors have reported inter-and intra-observer agreement in the range of .30-.88 [16-25] and .70-.94 [16-20], respectively, for populations of LBP patients [18,20-23], unspecified patients [17,24], asymptomatic patients [25] and general populations [16,19]. Of these studies, four report confidence intervals [16-19] thereby allowing reliable comparison of results between studies. In relation to the evaluation of the type of signal changes, two of the four studies reporting CIs had statistically higher inter-observer agreement [16,19] and two had comparable results [17,18]. The intra-observer agreements found in all four studies were comparable with the results from the current study. However, both study samples and the definitions of signal changes in these studies differed from the current study. Agreements regarding location of signal changes were reported in two of the studies [16,19] and were in concordance with the current study; however these definitions also varied from the one used in the current study. Agreements regarding size of signal changes were reported in one of the four studies, with results in concordance with the current study [16]. In relation to the evaluation of signal changes located in the vertebral corner, agreement of BMO and FMD corner lesions has been analysed in a previous study sample encompassing 20 patients with established SpA. The reported kappa values ranged from 0.23 to 0.72 for BMO lesions [26] and from 0.60 to 0.72 for FMD lesions [14]. However, differences in the definitions and in the study sample preclude a direct comparison of results.

Disc degeneration was assessed using Pfirrmann’s grading system [27] and substantial to almost perfect inter- and intra-observer agreements, respectively, were found in accordance with earlier reports on this grading system [27,28], although no studies with CIs were identified. In relation to disc contour, similar agreements were found which are also comparable with previous reports [29,30].

The inter-observer agreement for herniations in the vertebral endplate was found to be fair. This is slightly inferior to the results of a previous study on LBP patients, but the intra-observer agreements were comparable [31].

In relation to Scheuermann’s changes, the inter-observer agreement was slight and the intra-observer agreement, moderate. To our knowledge, there are no previous agreement studies regarding Scheuermann’s changes using MRI.

In relation to the evaluations of the SIJ, either substantial or almost perfect inter- and intra-observer agreements were found for the majority of MRI changes in the current study. The exceptions were for the intensity of BMO, the depth of FMD and erosions which had a moderate inter-observer agreement. To our knowledge, no earlier studies report on the agreement of these changes assessed as single lesions. Several studies that assess each lesion individually were identified. However, these studies report only results on analysis performed on combinations of these findings, e.g. sum score of total findings or anatomical regions [32-36], which are not comparable with assessing agreement on each lesion.

Regarding global assessment, one recent study investigated the inter-observer agreement for global evaluation of MRI of the SIJ in SpA versus non-SpA patients. The kappa value for inter-observer agreement for 5 categories of confidence in the SpA diagnosis were found to be .73 (.62-.81) in a cohort of back pain patients referred to a secondary care outpatient clinic in Switzerland due to suspicion of SpA and .74 (.65-.80) in cohorts of back pain patients with anterior uveitis referred to a ophthalmology department in Canada [37]. This is higher than the inter-observer agreement found for global assessment in the current study but with overlapping CI. In general, the spinal MRI findings related to SpA are not as clearly defined as the findings related to the SIJ, which is reflected in the incorporation of only SIJ changes in the ASAS criteria for SpA. Therefore, one reason for the lower agreement in the current study could be that the inclusion of spinal changes in the global assessment increases the uncertainty of the diagnosis.

### Application of the findings

The acceptable agreement for the evaluation of key MRI changes in the spine and SIJ makes it possible to use these MRI changes in the BaPa Cohort study and other studies investigating MRI changes in patients with non-specific LBP and suspected SpA.

Earlier publications on the evaluation of SpA-related MRI findings have mainly been focused on grading systems for active and chronic SpA changes as a measurement of disease severity in already diagnosed SpA patients. However, the assessment of each lesion separately creates the potential for additional analysis of the diagnostic and prognostic value of each individual MRI finding. It also creates the potential for describing the development of the changes in subsequent longitudinal studies and it provides a possibility for analysing location-specific alterations, e.g. to compare MRI changes with pain location. Furthermore, the inclusion of both SpA-related and degenerative changes in the same evaluation protocol facilitate an accessible assessment of MRI findings that could mimic SpA-related findings, assessed under the same standardized evaluation session.

### Strengths and weaknesses of the study

This study has potential weaknesses that have to be addressed. Firstly, some MRI changes could not be analysed because of too few positive ratings, and the agreement of the evaluation of these findings could not be tested. If this problem were to be addressed, the study population would have to have contained patients with more pronounced SpA. However, this would have made the study sample less applicable to the BaPa Cohort, to which the evaluation protocol will be applied. For some of the MRI changes, the inter-observer agreement varied between reader pairs, despite training and calibration sessions, indicating that more effort could have been done in calibration, especially regarding vertebral disc herniation and Scheuermann’s changes.

This study also has a number of strengths. MRI of the whole spine and SIJ were read by three independent readers and intra-observer agreement was tested by two of the readers. The involvement of more than two readers improves the generalisablity of the evaluation method. Moreover, for the MRI changes related to SpA in both the spine and SIJ, this is the first time agreement has been tested assessing each lesion separately. This creates the potential for describing the development of the changes in subsequent studies, and the possibility for analysing location-specific alterations. Furthermore, the readers were highly specialized musculoskeletal radiologists, and training and calibration sessions were conducted prior the readings.

## Conclusion

The inter- and intra-observer agreement for the evaluation of spondyloarthritis-related and degenerative MRI changes in the spine and spondyloarthritis-related changes in the sacroiliac joints were investigated in this study. In the spine, substantial to almost perfect observer agreement was found for the evaluation of the location and the size of vertebral signal changes and for disc degeneration and disc contour. In the sacroiliac joints substantial to almost perfect observer agreement was found for the grading of bone marrow oedema and fatty marrow deposition, the depth of bone marrow oedema and for subchondral sclerosis. Also, ‘Global assessment’ regarding the spondyloarthritis diagnosis had substantial or almost perfect observer agreements.

## Abbreviations

SpA: Spondyloarthritis; SIJ: Sacroiliac joints; MRI: Magnetic resonance imaging; BaPa Cohort: The Back Pain Cohort of Southern Denmark; DVU: Discovertebal unit; CI: Confidence interval; BMO: Bone marrow oedema; FMD: Fatty marrow deposition.

## Competing interests

The authors declare they have no competing interests.

## Authors’ contributions

BA, TSJ, CM, and AGJ contributed to conception and design of the study. AZ, NE and AGJ evaluated the MRI scans. BA performed the interpretation and analyses of data and drafted the manuscript. TSJ participated in the interpretation and analyses of data and helped draft the manuscript. AGJ helped draft the manuscript. All authors read, critical reviewed and approved the final version to be submitted for publication.

## Pre-publication history

The pre-publication history for this paper can be accessed here:

http://www.biomedcentral.com/1471-2474/14/274/prepub

## Supplementary Material

Additional file 1 “Definitions of the MRI changes assessed in the spine and sacroiliac joints”. Click here for file
