# Supplementary material for: Spondyloarthritis-related and degenerative MRI changes in the axial skeleton - an inter- and intra-observer agreement study
Source: BMC Musculoskelet Disord. 2013 Sep 23;14:274. doi: 10.1186/1471-2474-14-274 (PMC3848902; doi:10.1186/1471-2474-14-274)
Supplement: Additional file 1 — “Definitions of the MRI changes assessed in the spine and sacroiliac joints”. [file 1471-2474-14-274-S1.doc]

**Definitions of MRI changes assessed in the spine and sacroiliac joints**

**The spine**

| **Variable** | **Definitions** |
| --- | --- |
| **Type of signal change** | BMO: A subchondral area with increased signal on STIR images.  FMD: A subchondral area with increased signal on T1-weighted images compared with normal bone marrow.  Mixed lesion: A subchondral area with increased signal on both T1-weighted and STIR images. |
| **signal change in the corner** | A lesion which is primarily located in the corner of the vertebra and has contact with both edges of the corner. Changes in relation to osteophytes are not scored as corner lesions. |
| **Size of signal change in the vertebral endplate** | The size is evaluated from the area of the endplate.  Definition of 100%: The entire subchondral area corresponding to the endplate. I.e. the depth of the lesion is not important.  Small: <25% of the subcortical bone area.  Medium: 25% - <50% of the subcortical bone area.  Large: ≥50 % of the subcortical bone area. |
| **Location of signal change** | Anterior localisation: >50 % of the lesion is situated in the anterior part of the vertebrae.  Posterior localisation: >50 % of the lesion is situated in the posterior part of the vertebrae.  Equally widespread: The lesion is equally distributed with approximately 50% of the lesion in the anterior and the posterior part of the vertebra, respectively. |
| **Total size of BMO in the DVU** | The total size of all the BMO lesions in the DVU. The size of each lesion is furthermore evaluated separately in the variables above.  Small: <25% of the subcortical bone area.  Medium: 25% - <50% of the subcortical bone area.  Large: ≥50 % of the subcortical bone area. |
| **Total size of FMD in the DVU** | The total size of all FMD lesions in the DVU. The size of each lesion is furthermore evaluated separately in the variables above.  Small: <25% of the subcortical bone area.  Medium: 25% - <50% of the subcortical bone area.  Large: ≥50 % of the subcortical bone area. |
| **Total size of mixed lesions in the DVU** | The total size of all mixed lesions in the DVU. The size of each lesion is furthermore evaluated separately in the variables above. Only lesions evaluated as mixed lesions should be included in this evaluation.  Small: <25% of the subcortical bone area.  Medium: 25% - <50% of the subcortical bone area.  Large: ≥50 % of the subcortical bone area. |
| **Erosions** | Loss of normal marrow signal on T1 with a defect in the overlaying cortical bone.  Slight: Erosions covering <25% of the vertebral plate.  Moderate: Erosions covering 25% to <50% of the vertebral plate.  Severe: Erosions covering ≥50% of the vertebral plate. |
| **Erosions in the corner** | Evaluated as located in the corner if a minimum of one of the erosions is located in the corner. |
| **Syndesmophytes or vertebral fusion** | Syndesmophytes are new bone formations from the edge of the vertebra. Spondylophytes and ambiguous syndesmophytes with a growth angle of >45 degrees in relation to the anterior vertebral edges should not be considered as syndesmophytes.  Slight: Syndesmophytes between vertebral corners without osseous bridging.  Moderate: Syndesmophytes and partial osseous bridging between the vertebral bodies.  Severe: Total fusion of two vertebral bodies. |
| **BMO at the costovertebral joints** | An osseous area laterally in the thoracic vertebral bodies with increased signal on STIR images. |
| **BMO at the apophyseal joints** | Subchondral areas at the apophyseal joint with increased signal on STIR images. |
| **FMD at the apophyseal joints** | Subchondral areas at the apophyseal joint with increased signal on T1-weigthed images compared with normal bone marrow. |
| **Soft tissue oedema** | An area with increased signal on STIR images in the soft tissue at the apophyseal joints, excluding synovial cysts regarded as degenerative. |
| **Disc degeneration** | Normal: Normal height and signal intensity in the disc.  Slight: A slight decrease in height and/or signal intensity in the disc.  Moderate: Decreased height and fluid signal in the disc. Severe: Elimination of the disc height.  NB: High intensity in the disc due to blood vessel ingrowths is noted in the comment box. |
| **Disc contour** | Broad-based protrusion: Herniation involves 25-50% of the disc circumference.  Focal protrusion: Herniation involves 0-25% of the disc circumference.  Extrusion: Herniation is longer than it is broad or migration of the herniation over or under the level of the disco-vertebral corners.  Sequestration (free fragment): The herniation fragment is without communication with the disc. |
| **Disc herniation in the vertebra endplate** | Localised irregularity of vertebral endplate with persistent cortical demarcation of > 3 mm in depth. |
| **Scheuermann’s changes** | Disc herniation in the vertebral endplate or at the vertebral corners at several levels (not necessarily 3 consecutive DVUs). There may be associated BMO, FMD and disc degeneration. These changes should also be evaluated under the respective variables. |
| VE: Vertebral endplate, DVU: disco vertebral unit, STIR: Short-tau inversion recovery, BMO: bone marrow oedema, FMD: Fatty marrow deposition | |

**The sacroiliac joints**

| **Variables** | **Definitions** |
| --- | --- |
| **BMO** | A subchondral area with increased signal on STIR images. The lesion has to be present at 2 places in 1 slice or at 1 place in 2 consecutive slices.  Definition of 100%: The entire subchondral area corresponding to the joint facet in the region is affected. I.e. the depth of the lesion is not important.  Slight: <25% of the subcortical bone area.  Moderate: 25% - <50% of the subcortical bone area.  Severe: ≥50% of the subcortical bone area. |
| **Intensity of BMO** | Normal to moderate: Normal to moderately increased intensity.  Pronounced increased: Signal intensity comparable with that of the spinal fluid and covering an area of ≥1cm^2^. |
| **Depth of BMO** | Normal to moderate: Oedema extending <1 cm beneath the joint surface.  Widespread: Oedema extending ≥1cm beneath the joint surface and covering ≥1 cm^2^.  If the entire width of the ilium is affected, the score is always “widespread”. |
| **FMD** | A subchondral area with increased signal on T1-weigthed images compared with normal bone marrow. The lesion has to be present at 2 places in 1 slice or at 1 place in 2 consecutive slices.  Definition of 100%: The entire subchondral area corresponding to the joint facet in the region is affected. I.e. the depth of the lesion is not important.  Slight: <25% of the subcortical bone area.  Moderate: 25% to <50% of the subcortical bone area.  Severe: ≥50% of the subcortical bone area. |
| **Depth of FMD** | Normal to moderate: FMD extending <1 cm beneath the joint surface.  Pronounced: FMD ≥1 cm beneath the joint surface and covering ≥1 cm^2^.  If the entire width of the ilium is affected, the score is always “widespread”. |
| **Subchondral sclerosis** | A subchondral area with low signal, compared to normal bone marrow on T1, T1FS (SPIR) and STIR images. The lesion has to be present at 2 places in 1 slice or at 1 place in 2 consecutive slices.  Slight: <25% of the subcortical bone area.  Moderate: 25% - <50% of the subcortical bone area.  Severe: >50 % of the subcortical bone area. |
| **Erosions** | Loss of marrow signal on T1 and T1FS with a defect in the overlaying cortical bone (often best delineated at T1FS). For subtle changes, erosion has to be present at 2 places in 1 slice or at 1 place in 2 consecutive slices.  Definition of 100%: The entire subchondral area corresponding to the joint facet in the region is affected.  Slight: Erosions covering <25% of the joint facet area.  Moderate: Erosions covering 25% to <50% of the joint facet area.  Severe: Erosions covering ≥50% of the joint facet area. |
| **Ankylosis** | Partial: Partial osseous bridging across the joint space.  Total: Fusion of the joint facets. |
| **Global assessment** | Based on the entire MR examination, how strongly do you agree to the following: "This patient has SpA”?  Strongly disagree  Disagree  Neither agree or disagree  Agree  Strongly agree |
| BMO: Bone marrow oedema, FMD: Fatty marrow deposition, STIR: Short-tau inversion recovery, FS: Fat saturated | |
